# Mapping the legal foundations of planetary mental health

**DOI:** 10.1017/gmh.2022.22

**Published:** 2022-04-18

**Authors:** Eric C. Ip, Daisy Cheung

**Affiliations:** Centre for Medical Ethics and Law, The University of Hong Kong, Pokfulam Road, Hong Kong, Hong Kong SAR

## Mental well-being in the Anthropocene

Human health hinges on the integrity of the planetary biophysical environment and local ecosystems (Zywert and Quilley, 2020). Climate change, ocean acidification, freshwater supplies overconsumption, air pollution, stratospheric ozone depletion, and toxic waste have triggered extreme weather events, rising temperatures, biodiversity loss, food degradation, and new communicable diseases that have severely impacted on not only ecosystems (Phelan, 2020), but also the physical and mental health of children, the elderly, and other vulnerable populations with pre-existing chronic health conditions and low economic status (Ingle and Mikulewicz, 2020).

Mental disorders associated with climate change are far less visible than physical ones, particularly in low- and middle-income countries, in which public mental healthcare tends not to be prioritized by international development agencies and national governments. Extraordinary heat events noticeably increase hospital admissions for behavioral and mood disorders such as mania, neurotic disorders, and schizophrenia (Hayes *et al*., 2018). Extreme weather events such as flooding, hurricanes, tsunamis, and wildfires can lead to acute shock, increased incidence of suicide and suicidal ideation, and post-traumatic stress disorder (PTSD); gradual climate development like changes in temperature patterns, increased frequency of droughts, disappearance of rivers, and rising sea levels can alter the social determinants of mental health (Chan, 2020), and result in chronic psychological distress and anxiety in the long run (The Lancet Planetary Health, 2017). Widespread ecological degradation and abject governmental responses to food and water insecurity additionally contribute to the occurrence of armed conflict and civil unrest (Hayes *et al.*, 2018), which are likely to result in adverse psychological effects in affected populations.

Furthermore, the untenability of local environmental conditions may cause the permanent mass displacement of sizeable populations because of the disruption of communities and destruction of homes (Lemery and Auerbach, 2017), which, in turn, can bring forth significant psychosocial stress (Cianconi *et al.*, 2020). The scarcity of clean water and other necessities of life due to pollution and climate change is regarded in many cultures as a source of shame and humiliation, which can turn family members and neighbors against each other (Wutich *et al.*, 2016). Alarmingly, people experience pre-traumatic stress disorder and climate distress in anticipation of planetary and environmental changes as well (Clayton, 2020). Ecoanxiety, characterized by debilitating, severe worry about climate risks, can result in a dramatic loss of appetite, insomnia, obsessive thinking, and panic attacks (Gifford and Gifford, 2016), which may amalgamate with other daily stressors to trigger anxiety disorders, depression, and substance abuse (Horton and Lo, 2015). In addition, climate change can aggravate preexisting inequities faced by persons with mental disabilities (United Nations Human Rights Council, 2020).

In response to the alarming impact climate change has had on human health, the field of planetary health has emerged in recent years, focusing on ‘the achievement of the highest attainable standard of health, wellbeing, and equity worldwide through judicious attention to the human systems—political, economic, and social—that shape the future of humanity and the Earth's natural systems that define the safe environmental limits within which humanity can flourish’ (Whitmee *et al.*, 2015). This field has thus far focused primarily on physical health, however, and given that there can be no health without mental health (Prince *et al.*, 2007), much more emphasis needs to be placed on the under-researched area of planetary mental health (Jevtic and Bouland, 2019). Importantly, it should be noted that the crisis of planetary mental health forms part of the global mental health crisis (Ip and Cheung, 2020), in which major depressive disorders have become the second leading cause of disability worldwide and affect population health to a greater extent than coronary artery disease and diabetes (Williams, 2019).

## The concept of planetary mental health law

The conceptualization of planetary mental health should have as its starting point the clear recognition that climate change is a cause of a myriad of population-wide mental health problems across many parts of the world. Law has a role to play in obligating state actors to address the health consequences of the climate crisis (Ganesh *et al.*, 2020). In delineating the kinds of obligations that states have to address this issue, it should be noted that there is currently no dedicated legal regime for either planetary health or planetary mental health. We take the view, however, that the legal principles governing planetary mental health already exist, although they are in need of further development and strengthening.

While what we call ‘planetary mental health law’ differs from the emerging field of public mental health law (Coggon and Laing, 2019) in that it focuses specifically on mental health effects as a result of state inaction or inappropriate state action in addressing climate change, public mental health law does provide promising intellectual resources for teasing out the implications of existing international law instruments on planetary mental health. In particular, it maps the distinct legal obligations of the state to assure the very conditions necessary for people to attain and maintain mental health (Gable and Gostin, 2009), examples of which may include, within its available resources, the provision of providing decent working conditions, social and welfare services, primary and secondary mental healthcare, community and hospital-based mental health services, and so on (Cheung and Ip, 2020).

## Protecting planetary mental health with human rights and climate law

Although leading mental health-related treaties such as the International Covenant on Economic, Social and Cultural Rights (ICESCR) make no mention of climate change or planetary mental health, Article 12 of the ICESCR echoes the central concerns of this area by requiring states to take steps that are necessary for ‘[t]he improvement of all aspects of environmental and industrial hygiene’ and ‘[t]he creation of conditions which would assure to all medical service and medical attention in the event of sickness’, with references to health here encompassing mental health. Another instrument that has important implications for planetary mental health is the UN Convention on the Rights of Persons with Disabilities (CRPD), the first human rights treaty of the twenty-first century and one that definitionally includes individuals with mental impairments (Bartlett, 2012). Article 11 of the CRPD states that member-states should take ‘all necessary measures to ensure the protection and safety of persons with disabilities in situations of risk, including … the occurrence of natural disasters’. Apart from international treaties, soft law documents are also relevant. Sustainable Development Goal 3.4, embodied in ‘The 2030 Agenda for Sustainable Development’ adopted by the UN General Assembly in 2015, states member-states' commitment to ‘promote mental health and well-being’ in the context of reducing one-third premature mortality by 2030, and to ‘take urgent action to combat climate change and its impacts’.

The right to mental health, enshrined in Article 12 of the International Covenant on Civil and Political Rights (ICCPR), obligates states, subject to their available resources, to achieve ‘full realization’ of the ‘highest attainable standard’ of mental health; this has been taken to include the obligation to safeguard ‘the conditions necessary for people to attain and maintain mental health’ (Gostin and Gable, 2004). A right to planetary mental health can be inferred from the right to mental health, if these conditions to be safeguarded are understood as including healthy environmental conditions that make clean drinking water possible and environmentally-driven displacement unnecessary. Fewer mitigatable or adaptable natural disasters will also likely mean fewer incidences of PTSD (Cianconi *et al.*, 2020). It is well known that exposure to clean green and blue areas, as a result of successful environmental action, is associated with enhanced mental wellbeing (de Keijzer *et al.*, 2019), possibly through causal mechanisms such as the replenishment of cognitive capacities, reduction of stress, and intensification of physical activity and social cohesion, all of which benefit mental health (Bratman *et al.*, 2019). Environmental conditions beneficial to mental health cannot be safeguarded without resolute action against anthropogenically induced climate change and environmental degradation.

The legal principles set out above have two key implications in terms of state obligations. First and foremost is that states need to take action to combat climate change to fulfil both their obligations under international environmental law and international human rights law. This should be reflected in any framework or treaty that places obligations on states to address climate change, such as the UN Framework Convention on Climate Change (UNFCCC), which needs to have, as one of its explicit and fundamental goals, the protection of the right to health, including mental health. The UNFCCC, in Article 2, merely states ‘the prevention of dangerous anthropogenic interference with the climate system’ as its ‘ultimate purpose’. The preamble of the Paris Agreement of 2015 commendably broke new ground in affirming that member-states, ‘when taking action to address climate change’, should ‘respect, promote and consider their respective obligations on human rights’, including ‘the right to health’, and the rights of ‘persons with disabilities and people in vulnerable situations’. Here it should be noted that the right to health should not simply be read as a right to be healthy, but rather ‘a right to access to a system of health protection which provides equality of opportunity for people to enjoy the highest attainable level of health’ (Haines and Frumkin, 2021). A central purpose of climate action, however, is to restore and protect the health of the planet and that of its human inhabitants as one integral whole, and while the mention of ‘the right to health’ points in the correct direction, it arguably does not go far enough because it fails to sufficiently recognize the importance of this right to health (both physical and mental) by expressly positioning it as a *goal* of climate action itself. Awareness of the inherent importance of the right to health as a goal of climate action, and of climate action as a means to protecting the right to health, needs to be firmly underscored and recognized.

It should be noted that in their pursuit of this goal, it is key that states do not overlook the potential of mandatory measures to have a disproportionately large and often adverse effect on the underprivileged. It has been suggested that environmentally friendly choices are often unaffordable for low-income households (Brown *et al.*, 2020). If the use of environmentally friendly materials were to be made compulsory and the cost were to be borne by individuals or families with variable means to pay for such materials, this would arguably be an extremely regressive policy with negative health and economic consequences, such as additional, and in some cases unaffordable, financial burdens that would, in turn, become a source of significant stress and anxiety. It is in many ways also unwise for policymakers to resort to such individually-focused policies, as these are unlikely to yield holistic changes to social systems and structures that would be likely to result in greater influence. In devising and implementing measures to combat climate change, states must ensure that the measures themselves do not result in preventable hazards to public mental health, thereby violating the right to mental health, as alluded to in the Preamble of the Paris Agreement, which recognizes that parties may be affected by ‘the impacts of the measures taken in response to [climate change]’.

Second, states should take steps to address mental health problems inflicted by climate change and associated extreme weather events and natural disasters. In the Global North, this will include the enhancement of service delivery to meet the increase in demand resulting from what will likely be a significantly higher level of mental morbidities, which in most cases will require careful policy planning and a higher government expenditure on mental health (Park *et al.*, 2020), given the imbalance that often already exists between mental health budgets and mental health disease burdens (Vigo *et al.*, 2019). In the Global South, wherein the above measures are unlikely practicable, attention should be paid to how low-income settings or local cultures might play an invaluable role in coming up with solutions. Indigenous communities, for example, often possess unique spiritual, cultural, and legal connections to their land and environment (Warner, 2015). Indigenous-led renewable energy projects in Canada have reduced reliance on fossil fuels and fostered a wider and more intergenerational distribution of the fruits of energy generation, both of which have been highly beneficial to developing a reciprocal form of ecological stewardship (Smith and Scott, 2021). The power of such indigenous wisdom in empowering people to identify some of the best solutions to planetary mental health problems should therefore not be underestimated.

## Harnessing the power of soft law for planetary mental health

Despite the existence of legal principles that can be interpreted as imposing state obligations in relation to planetary mental health law, the current state of affairs remains deeply unsatisfactory. To harness the full power of law (Gostin *et al.*, 2019) for planetary mental health, we believe it is necessary to overcome the ubiquitously fragmented architecture of laws relating to Earth governance (Biermann, 2014). We propose that the following steps be taken. First, the relationship between climate change and mental morbidities needs to be brought to the forefront of international attention. This can and should be done via two methods, the first being explicit references in key treaties. For instance, if a Framework Convention on Global Health is ever adopted (Gostin, 2016), it should carry with it provisions that directly address the needs of planetary mental health. Leading climate treaties should be amended to feature rights to health and healthy environments as explicit goals of climate action. Although we have argued above that the key health-related treaties already contain legal principles which translate into state obligations to protect public mental health, these treaties should also be modified to contain equally explicit references to the environmental crisis. We do recognize, however, how hard it can be to amend international conventions. Until amendments could be achieved, the Conference of the Parties, international organizations and tribunals, national authorities, and other relevant actors can borrow from the interpretive doctrine of ‘living instrument’ established in the jurisprudence of UN human rights bodies and the European Court of Human Rights (Fitzmaurice, 2013, p. 767), to construe the UNFCCC, Paris Agreement, and so on, as organically growing instruments that address the ever-changing circumstances of our planet. This would enable key actors in planetary health governance to make explicit implicit principles within these treaties that are protective of the mental health of present and future generations.

The second method, closely related to the first, is through the creation of new soft law measures by global health actors and national governments, aimed at tightening the overall coherence of the emerging planetary mental health legal regime, connecting isolated provisions on health protection or climate governance dispersed in various treaties in the form of a WHO normative framework like the Pandemic Influenza Preparedness Framework, or a UNFCCC Conference of the Parties declaration. Soft law can also be drafted to provide relevant provisions with new interpretations that explicitly point toward planetary mental health objectives – for instance, a new Comment of the Committee on Economic, Social and Cultural Rights that authoritatively reinterprets Article 12 of the ICESCR as accommodative of the demands of climate action. Although much important work has been going on within WHO, the Organization has yet to produce a soft law document that specifically addresses the right to mental health in the context of climate change, let alone promulgate a resolution of the World Health Assembly that directly teases out the implications of the right to a healthy planet and how this is conducive to mental health. It is telling that the term ‘mental health’ appeared merely once – and not in its human rights sense – in the 2020 WHO Global Strategy on Health, Environment and Climate Change, which runs 36 pages.

The development of planetary mental health soft law may not be as difficult as one might imagine, because soft law already constitutes the bulk of global health law, as a widely used complement to treaties (Hodge *et al.*, 2020). Although soft laws may not initially appear to be credible international commitments (Guzman, 2008), some soft law principles do eventually go on to become ‘candidates for eventual recognition as fully fledged law’ (Charlesworth, 2012). In addition, soft law instruments are far less costly to negotiate, and thus also flexible enough to adapt to rapidly changing circumstances (Sekalala, 2017), be it human-induced climate change, or its dire consequences on human mental health.

Third, national governments should amend their domestic constitutions or other fundamental laws to enshrine credible commitments to protecting planetary health (Ip and Lee, 2021), including planetary mental health. In pursuing actions aimed at combating climate change, these governments must take into consideration their public mental health obligations when devising and implementing relevant measures. To fulfill their duties to guarantee the right to public mental health in a planetary health context, domestic authorities can plan ahead of extreme weather events to buttress their own adaptation and resilience capacities, administer mental health epidemiological surveys in the aftermath of disasters, and fashion more public mental health agencies that specialize in providing people with psychological first aid, as well as treatments beneficial to psychosocial wellbeing (Hayes *et al.*, 2018). To entrench these commitments, states should consider constitutionalizing them, or at least legislating them into framework statutes that address planetary mental health emergencies.

Given the multifarious difficulties of amending national constitutions, domestic high courts should actively explore how a right to planetary mental health can be read into existing public law through interpretive tools like the ‘living instrument’ doctrine mentioned above. Additionally, they may draw analogies from the landmark 2019 decision of the Dutch Supreme Court in *State of the Netherlands v. Urgenda Foundation*, which upheld the state's positive obligation to reduce its greenhouse gas emissions by no less than 25% from its 1990 level by the end of 2020, in order to safeguard the right to life guaranteed by the European Convention on Human Rights (Meguro, 2020). If the right to health is an indispensable facet of the right to life, and if the right to mental health is an indispensable facet of the right to health, then the right to mental health must be an indispensable facet of the right to life.

## Concluding remarks

The recent signs of the possible collapse of the Gulf Stream suggest that irreversible changes to the environment are becoming increasingly likely (Gonçalves Neto *et al.*, 2021). Such drastic developments in the climate crisis point to correspondingly dire consequences to the socioeconomic determinants of the mental health of populations. The mental health burden unleashed by climate change is disproportionately borne by the marginalized and low-income populations who are short of resources to support themselves (Abdalla *et al.*, 2021). In the Indo-Pacific region, for example, the mass displacement of ‘climate refugees’ from countries that are facing existential threats, such as the Maldives and Tuvalu to Australia and New Zealand, respectively, will inevitably trigger assimilation difficulties in addition to the trauma associated with being forced to leave their home countries, which in turn, may materialize into cultural anxiety, depression, PTSD, and sometimes also suicidal ideation (Lemery and Auerbach, 2017).

We observed that legal principles relevant to planetary mental health are currently scattered across the discrete bodies of human rights, health, and climate law, each of which go some way in creating twofold state obligations to take climate action for the purpose of attaining health and thereby mental health, and address mental morbidities resulting from the climate crisis. We thus argued that a ‘planetary mental health law’ already exists, albeit in preliminary form, and in great need of being developed into a clearer and more coherent regime. Prior to proposing fundamental changes to the international system, it appears to us that soft law should be resorted to as a complement to existing international obligations, whereas states should bolster their level of national commitment to defend the right to mental health on a healthier planet. Time is not on our side and the law must act now.
